# Safflower Protein Hydrolysates: Physicochemical, Functional Properties and Antioxidant Activities

**DOI:** 10.1002/fsn3.70258

**Published:** 2025-05-11

**Authors:** Fatma Korkmaz, Ceren Mutlu

**Affiliations:** ^1^ Food Engineering Department, Engineering Faculty Balıkesir University Balıkesir Türkiye; ^2^ Centro de Investigacão de Montanha (CIMO) Instituto Politécnico de Bragança Bragança Portugal

**Keywords:** Alcalase, degree of hydrolysis, enzymatic hydrolysis, Flavourzyme, safflower protein

## Abstract

This study aimed to investigate the effect of enzymatic hydrolysis on the physicochemical, functional, and antioxidant properties of safflower protein isolate and hydrolysates. Isolated safflower protein was hydrolyzed by both Alcalase and Flavourzyme at the degree of hydrolysis of 2%, 4%, 6%, 8%, and 10%. Safflower protein hydrolysates exhibited a lighter color (3.74%–8.79%) and reduced redness (69.11%–102.85%) with lower cohesiveness (15.29%–21.76%) and better flowability (25.91%–40.27%) compared to the protein isolate. Moreover, the surface hydrophobicity of safflower protein isolate decreased up to 73.18% with hydrolysis, while solubility increased up to 54.42% at pH 4–7. Safflower proteins hydrolyzed with Alcalase had higher oil binding, foaming, and emulsion capacities than samples hydrolyzed with Flavourzyme, while their water holding capacities were lower. Furthermore, safflower proteins hydrolyzed with Alcalase at an 8% degree of hydrolysis displayed the highest foaming capacity (up to 3.89 times) and emulsion capacity (up to 1.23 times) in all samples. However, it had poor foam (up to 67.06%) and emulsion stability (up to 74.35%). Additionally, safflower protein hydrolysates demonstrated higher ABTS^•+^ and DPPH radical scavenging activity. Overall, safflower protein hydrolysates showed better physicochemical, functional, and antioxidant properties than protein isolates, depending on enzyme types and degree of hydrolysis.

## Graphical abstract

This study investigated how enzymatic hydrolysis with Alcalase and Flavourzyme at different DH levels affected the powder, functional, and antioxidant properties of safflower protein isolate. Depending on the type of enzyme and the DH level, hydrolysis decreased cohesiveness and increased flowability, improved solubility, reduced surface hydrophobicity, and enhanced foaming and emulsifying capacities. Additionally, hydrolyzed proteins exhibited stronger antioxidant activity.

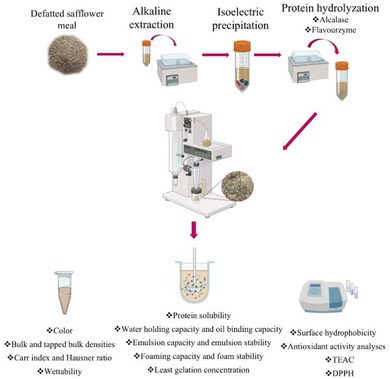

**Abbreviations**
BPB: bromophenol blue
EC: emulsion capacity
ES: emulsion stability
FC: foaming capacity
FS: foam stability
LGC: least gelation concentration
OBC: oil binding capacity
PS: protein solubility
SPH: safflower protein hydrolysate
SPHA: Alcalase‐treated safflower protein hydrolysate
SPHF: Flavourzyme‐treated safflower protein hydrolysate
SPI: safflower protein isolate
WHC: water holding capacity

## 1 Introduction

Safflower ( *Carthamus tinctorius* L.) is an oilseed plant known for its adapting to various climatic and agronomic conditions. Its seeds contain 15%–45% oil, depending on the variety, making it valuable for multiple industrial applications, including pharmaceuticals, cosmetics, and biodiesel production (Yıldırım and Çantaş 2023). Although safflower is primarily cultivated for oil extraction, the residual meal is rich in protein. Studies have reported that safflower meal contains about 20%–25% crude protein (Ustaoglu Tiril and Kerim 2015), which can increase up to 50% when decorticated (Galicia‐González et al. 2010). Due to its high protein content and functional properties, safflower meal is considered a promising alternative protein source (Yang et al. 2024). Studies suggest that isolated safflower proteins can be incorporated into various food products, including doughs, bakery items, soups, custards, ice creams, desserts, minced meat, and beverages (Barbhai et al. 2024). However, for plant‐derived proteins to be effectively used as substitutes in food applications, their functional properties, such as water and oil binding, foam and emulsion formation, stabilization, solubility, and gelation, must be improved. Additionally, undesirable characteristics, such as off‐flavors, bitterness, and anti‐nutritional factors, should be minimized (Nasrabadi et al. 2021).

Various techniques are employed to enhance the functional properties of plant proteins while reducing undesirable factors. These techniques include physical (e.g., heating, sonication, extrusion, ultrafiltration, radiation), chemical (e.g., acylation, glycation, deamidation, pH shifting), and enzymatic modifications. Physical modification methods are often energy‐intensive and costly, while chemical modification methods pose potential risks to public and environmental health due to the use of chemicals and the possible formation of toxic by‐products. In contrast, enzymatic modification is generally preferred due to its high specificity, short reaction time, mild conditions, minimal production of non‐toxic by‐products, controllable hydrolysis degree, cost efficiency, and environmental sustainability (Nasrabadi et al. 2021). Furthermore, enzymatic hydrolysis has been shown to improve the foaming and emulsifying capacities of proteins and may also enhance their bioactive properties, such as antioxidant activity and angiotensin‐converting enzyme inhibition (Wouters et al. 2016).

Enzymatic modification serves various purposes, including the removal of anti‐nutritional factors, structural modifications (e.g., phosphorylation, deamidation, cross‐linking), and protein hydrolysis (Arntfield 2018). Enzymes, such as Alcalase, Flavourzyme, Papain, Pepsin, and Trypsin, are commonly used for plant‐based protein modifications. The characteristics of protein hydrolysates vary depending on the mode of action of the enzyme used. For example, Alcalase is an endopeptidase that hydrolyzes proteins by cleaving internal peptide bonds, whereas Flavourzyme is an exopeptidase that catalyzes cleavage at the C‐ and N‐terminal ends of proteins. Both enzymes have been reported to enhance the functional properties, digestibility, and antioxidant activity of proteins at different levels. Additionally, Alcalase‐derived hydrolysates tend to exhibit a stronger bitter taste compared to those obtained with Flavourzyme, although they demonstrate superior antioxidative properties (Nasrabadi et al. 2021).

Enzymatic hydrolysis has been widely applied to improve the functional properties of various plant‐based proteins, including those from lentils (Vogelsang‐O'Dwyer et al. 2023), legumes (Xu et al. 2021), peanuts (Yadav et al. 2022), and peony seeds (Wang et al. 2021). To the best of our knowledge, there is no study investigating the enzymatic hydrolysis of proteins isolated from safflower meal and the changes in their functional properties. Therefore, this study aims to hydrolyze safflower meal‐derived proteins using Alcalase and Flavourzyme at varying degrees of hydrolysis to obtain protein hydrolysates with enhanced powder characteristics, functional properties, and antioxidant activity.

## 2 Materials and Methods

### 2.1 Materials

Safflower meal (variety Askon) was obtained by Ripsa‐Özşahin Tarımcılık (Kayseri, Türkiye). It was ground and sieved (1 mm) before the experiments and kept at 4°C. The Alcalase (Alcalase 2.4 L, protease from *Bacillus licheniformis* , ≥ 2.4 U/g) and Flavourzyme (Flavourzyme, protease from *Aspergilus oryzea*, ≥ 500 U/g) enzymes were supplied from Sigma‐Aldrich (Germany). Additionally, chemicals used in analyses were supplied as the analytical grade from Merck (Germany) and Isolab Laborgeräte GmbH (Germany) firms.

### 2.2 Alkaline Extraction and Isoelectric Precipitation

The protein extraction from the safflower meal was carried out according to the specified method (Korkmaz 2024). Before the protein extraction, safflower meal was defatted to increase protein extraction yield. For this aim, it was mixed with hexane (1:3, w/v) at 150 rpm (open‐air shaker OS‐4000, Jeio Tech, Korea) for 1 h at room temperature, and this process was repeated 3 times to remove all fat content from the meal. After this process, hexane was removed with filtration and drying of the defatted meal at room temperature for 18 h. It was kept at 4°C until the protein extraction (Korkmaz 2024).

Defatted safflower meal was dissolved in water, and a protein suspension (water:sample ratio 33.06:1) was prepared. The prepared suspension was extracted under the optimum conditions specified by Korkmaz (2024) as 23.3°C, pH 11.0 for 31 min. After extraction, centrifugation (NF 800, Nüve, Türkiye) was performed at 9.418 × *g* for 5 min to separate the precipitate from the supernatant, which was then filtered using filter paper. Then, the extracted protein fraction was separated by precipitation at the isoelectric point of pH 5.0. The excess water was removed from the precipitated protein fraction by centrifugation at 9.418 × *g* for 5 min. The moisture content of the separated protein fraction was determined by keeping it at 105°C until reaching constant weight. According to its moisture content, a protein suspension containing 10% dry matter was prepared with water, and the pH value was adjusted to 7.0 for the hydrolysis process.

### 2.3 Protein Hydrolyzation

The hydrolysis of the protein fraction of safflower meal was done at five degrees of hydrolysis (DH) as 2%, 4%, 6%, 8%, and 10% using either Alcalase or Flavourzyme enzymes. Additionally, the unhydrolyzed safflower protein isolate (SPI) was used as a control. In hydrolysis with Alcalase enzyme, the pH of the protein suspension was adjusted to 8.0, and the enzyme was added to the suspension with the enzyme:substrate ratio of 2%. The protein suspension was kept at 60°C in a water bath (ST30, Nüve, Türkiye) until the targeted DH was reached. In hydrolysis with Flavourzyme enzyme, the pH of the protein suspension was adjusted to 7.0, and it was kept at 50°C. The applied pH and temperature conditions were selected according to the optimum conditions where the enzymes show maximum activity. The pH values of the samples were controlled at certain intervals during enzymatic hydrolysis, and they were adjusted with 1 M NaOH. The consumed NaOH amount was used to determine the DH of the protein fraction according to the pH‐stat method specified by Adler‐Nissen (1986), and the DH of the protein fraction was calculated by the following equation.

$$\text{Degree of hydrolysis}\;\left(\%\right)=\frac{B\times C}{\alpha \times M\times h_{\mathrm{tot}}}\times 100$$

where *B* was the consumed NaOH volume (mL), *C* was the consumed NaOH concentration (M), *M* was the total amount of protein fraction (g), *h*_tot_ was the molecular weight number of peptide bonds in 1 g protein (mmol/g), and *α* was the average degree of decomposition of α‐NH_2_. The *α* value was calculated using the following equation (Rezvankhah et al. 2022).

$$\alpha =\left(10\;\mathrm{pH}-\mathrm{pK}\right)+\left(1+10\;\mathrm{pH}-\mathrm{pK}\right)$$

where pH was the value used in hydrolysis. pK represented the average pK value of the liberated α‐NH2 groups. pK was affected by temperature and was determined using the following equation.

$$\mathrm{pK}=7.8+\left(\frac{298-T}{298\times T}\right)\times 2400$$

where *T* was the temperature (Kelvin, K) at which enzymatic hydrolysis was maintained.

After reaching the targeted DH, the samples were kept at 85°C for 15 min to inactivate the enzymes. The samples were then cooled rapidly to room temperature, and pH values were adjusted to 7.0 before the spray drying process.

### 2.4 Drying Safflower Protein Hydrolysates

The safflower protein hydrolysates (SPH) and the SPI were dried in a spray dryer (B15 Mini Spray Drayer, Unopex, Türkiye). The drying was carried out according to optimized conditions as reported by Korkmaz (2024). The air inlet temperature of 160°C, aspiration rate of 54 m^3^/h, and feed flow rate of 16 mL/min were used for the spray drying of samples (Korkmaz 2024). The obtained powders were placed in sealed containers protected from air and light and kept at −20°C until analysis. The protein content of the samples was 97.60% on a dry basis. Photographs of the samples are presented in Figure S1.

### 2.5 Physicochemical Properties

#### 2.5.1 Surface Hydrophobicity

The surface hydrophobicity of samples was determined by preparing the 5 mg/mL protein powder solution in phosphate buffer (20 mM, pH 7.0) and mixing 3 mL of this solution with 600 μL of 1 mg/mL bromophenol blue. The prepared mixture stood for 10 min at room temperature and was centrifuged at 2.000 × *g* for 15 min. The supernatant of the samples was taken for determination of absorbance values in a spectrophotometer (UV‐1280 UV–VIS Spectrophotometer, Shimadzu, Japan) set to a wavelength of 595 nm. Additionally, phosphate buffer instead of sample solution was used as a control sample for the calculation of results by the following equation (Mutlu and Korkmaz 2024).

$$\begin{array}{c} \text{Bound bromophenol blue solution}\;\left(\mathrm{\mu g}\right)=\\ \frac{{\text{Absorbance}}_{\text{control}}-{\text{Absorbance}}_{\text{sample}}}{{\text{Absorbance}}_{\text{control}}}\times \left(200\;\mathrm{\mu g}\right) \end{array}$$

#### 2.5.2 Color

The L* [(0) black‐(100) white], a* [(+) red‐(−) green], and b* [(+) yellow‐(−) blue] color values of samples were measured from three different parts of the sample with a CR‐400 chromameter (Konica Minolta, Japan). Moreover, ΔE values were calculated using these color parameters.

#### 2.5.3 Bulk and Tapped Bulk Densities

The bulk density of the samples was determined by weighing approximately 2 g of sample into a 10 mL graduated cylinder and measuring its volume. The bulk density value was detected by measuring the volume after tapping the cylinder containing the sample 100 times on a flat surface. The results were given as the ratio of weight to detected volumes as g/cm^3^ (Akyüz and Ersus 2021).

#### 2.5.4 Carr Index and Hausner Ratio

The Carr index and Hausner ratio parameters were calculated by the following equations using the bulk density and tapped bulk density values (Akyüz and Ersus 2021; Özdemir et al. 2022)

$$\text{Carr index}\;\left(\%\right)=\frac{\rho_{\text{tapped}}-\rho_{\text{bulk}}}{\rho_{\text{tapped}}}\times 100$$

$$\text{Hausner ratio}=\frac{\rho_{\text{tapped}}}{\rho_{\text{bulk}}}$$

#### 2.5.5 Wettability

The wettability time was measured according to the reported method by Özdemir et al. (2022). According to the method, 0.1 g of the sample was poured into 100 mL of distilled water, and the time (s) for the sample to become completely wet was recorded.

### 2.6 Functional Properties

#### 2.6.1 Protein Solubility

For the determination of protein solubility (PS) of samples, a series of sample–water solutions were prepared at different pH levels between 2.0 and 12.0. Protein solutions were prepared by dissolving 0.2 g of sample in 20 mL distilled water, and pH values of solutions were adjusted with 1 M HCl or NaOH solutions. After that, the samples were incubated at room temperature for 30 min with stirring at 200 rpm. The centrifugation was done at 6.540 × *g* for 15 min to remove undissolved materials, and the supernatant was taken for analysis with the Lowry method (Lowry et al. 1951). The results were calculated with the following equation (Mutlu and Korkmaz 2024).

$$\mathrm{PS}\;\left(\%\right)=\frac{\text{Protein content in the supernatant}}{\text{Total protein content of sample}}\times 100$$

#### 2.6.2 Water Holding Capacity and Oil Binding Capacity

The water holding capacity (WHC) and oil binding capacity (OBC) of samples were determined according to the method reported by Korkmaz (2024). The WHC of samples was determined by mixing 0.25 g of sample with 5 mL of distilled water. After that, it was vortexed for 5 min and centrifuged at 5.000 × *g* for 10 min. For the weight detection of solids, the supernatant was removed. In the OBC analysis, 5 mL of sunflower oil was used instead of distilled water, and the analytical procedure was applied similarly. The results for each analysis were calculated using the following equation (Korkmaz 2024).

$$\mathrm{WHC}\;\text{or}\;\mathrm{OBC}\;\left(g/g\right)=\frac{W_{2}-W_{1}-W_{0}}{W_{0}}\times 100$$

The expressions of *W*_0_, *W*_1_, and *W*_2_ represent the initial dry weight of the sample, weight of the tube, and final tube and residue weight, respectively.

#### 2.6.3 Emulsion Capacity and Emulsion Stability

The emulsion capacity (EC) and emulsion stability (ES) of samples were determined with the reported method by Özdemir et al. (2022) with some modifications. For the determination of EC, protein powder solution (5%, w/v) was mixed with 10 mL of sunflower oil (1:1 v/v) and homogenized by ultra‐turrax (HG‐15D, Witeg, Germany) at 11.000 rpm for 30 s. After that, the obtained emulsion was centrifuged at 1.200 × *g* for 5 min. The volume of the formed emulsion layer and the total volume were noted to calculate EC using the following equation.

$$\mathrm{EC}\;\left(\%\right)=\frac{\text{Volume of emulsion layer}\;\left(\mathrm{mL}\right)}{\text{Total volume}\;\left(\mathrm{mL}\right)}\times 100$$

For the determination of ES, the prepared emulsions for the EC analysis were used, and tubes containing emulsions were kept at 80°C for 30 min in a water bath (ST 30, Nüve, Türkiye). After the incubation, samples were centrifuged at 1.200 × *g* for 5 min. The volume of the remaining emulsion layer was noted to calculate ES using the following equation.

$$\mathrm{ES}\;\left(\%\right)=\frac{\text{Volume of remaining emulsion layer}\;\left(\mathrm{mL}\right)}{\text{Volume of the orginal emulsion layer}\;\left(\mathrm{mL}\right)}\times 100$$

#### 2.6.4 Foaming Capacity and Foam Stability

The foaming capacity (FC) and foam stability (FS) of samples were determined according to the method reported by Kasapoğlu et al. (2021). For determination of FC, 20 mL of sample solution (1%, w/v) was homogenized by ultra‐turrax (HG‐15D, Witeg, Germany) at 12.000 rpm for 2 min. The volumes of the sample solution before and after whipping were measured and noted to calculate FC using the following equation.

$$\begin{array}{c} \mathrm{FC}\;\left(\%\right)=\\ \frac{\text{Volume after whipping}\;\left(\mathrm{mL}\right)-\text{Volume before whipping}\;\left(\mathrm{mL}\right)}{\text{Volume before whipping}\;\left(\mathrm{mL}\right)}\times 100 \end{array}$$

For the determination of FS, the prepared solutions for the FC analysis were used at room temperature for 30 min. After the duration, the solution volumes were noted to calculate FS using the following equation.

$$\mathrm{FS}\;\left(\%\right)=\frac{\text{Volume after duration}\;\left(\mathrm{mL}\right)}{\text{Initial foam volume}\;\left(\mathrm{mL}\right)}\times 100$$

#### 2.6.5 Least Gelation Concentration

For the determination of least gelation concentration (LGC) levels of samples, a series of sample solutions at different concentrations (2%–50%, w/v) were prepared with distilled water. These solutions were kept in a water bath at 100°C for 1 h to form a gel structure in samples. After that, the samples were immediately cooled in an ice bath and kept at 4°C for 2 h. The solution concentration at which the sample did not flow downwards when the tubes were inverted was determined visually and recorded as LGC (Sethi et al. 2021).

### 2.7 Antioxidant Activity Analyses

The antioxidant properties of samples were evaluated by ABTS^•+^ and DPPH radical scavenging activity analyses. For the analysis, 5 mg/mL of sample solution was prepared in distilled water. In the ABTS^•+^ radical scavenging activity analysis, 2.5 mL of ABTS^•+^ radical solution, prepared as described by Cui et al. (2022), was added to 20 μL of sample solution. This mixture was kept in a dark place for 6 min, and after that, the absorbance values were measured with the spectrophotometer set to a wavelength of 734 nm.

In the DPPH radical scavenging activity analysis, 1.5 mL of 0.2 mM DPPH radical solution was added to 1.5 mL of sample solution. This mixture was incubated in the dark at room temperature (25°C) for 30 min, and after that, the absorbance value was measured with the spectrophotometer set to a wavelength of 517 nm (Cui et al. 2022).

In both analyses, distilled water was used as a blank/control, and the results were calculated with the following equation.

$$\begin{array}{c} \text{ABTS or DPPH scavenging activity}\left(\%\right)=\\ \frac{{\text{Absorbance}}_{\text{blank}/\text{control}}-{\text{Absorbance}}_{\text{sample}}}{{\text{Absorbance}}_{\text{blank}/\text{control}}}\times 100 \end{array}$$

### 2.8 Statistical Analysis

The statistical analysis of the results was conducted by one‐way ANOVA using Minitab (ver. 17.0). Statistical differences between means were determined using Tukey's test, and the significant difference was defined as *p* < 0.05. All measurements were performed with at least three repetitions, and the results were reported as mean ± standard error based on dry matter content.

## 3 Results and Discussion

### 3.1 Physicochemical Properties

#### 3.1.1 Surface Hydrophobicity

**FIGURE 1:**
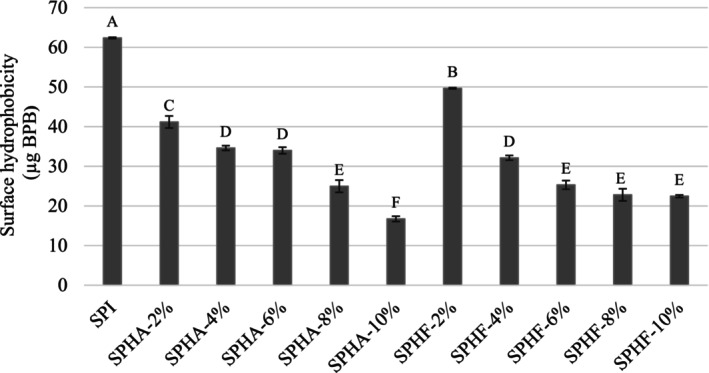
Surface hydrophobicity of safflower protein isolate and hydrolysates. *p* < 0.05 is statistically significant.

The surface hydrophobicity of proteins determines both the affinity and properties of protein–protein interactions. These hydrophobic interactions enable polypeptide chains to form globular structures and affect the functional properties of proteins (Yolandani, Liu, et al. 2024). The surface hydrophobicity of proteins is used to evaluate the functional properties, such as solubility and interfacial properties, due to their extreme sensitivity to protein hydrolysis (Liu et al. 2010; Vogelsang‐O'Dwyer et al. 2023). The surface hydrophobicity values of SPI and SPH are shown in Figure 1. The surface hydrophobicity of SPI was found to be 62.37 μg BPB, and it was significantly decreased after hydrolysis with both Alcalase and Flavourzyme (*p* < 0.05). Ren et al. (2017) reported a decrease (50%) in surface hydrophobicity of sunflower protein after hydrolyzation with Alcalase (10% DH). Vogelsang‐O'Dwyer et al. (2023) also found that the surface hydrophobicity of lentil protein decreased up to 31% and 7% with Alcalase (6.1% DH) and Flavourzyme (5.0% DH) hydrolysis, respectively. The decrease in surface hydrophobicity with hydrolysis may be due to the coming together of hydrophobic regions through hydrophobic interactions and the embedding of hydrophobic groups into the structure (Vogelsang‐O'Dwyer et al. 2023).

The surface hydrophobicity of SPHA decreased from 41.18 to 16.73 μg BPB with the increase of DH (*p* < 0.05). The surface hydrophobicity of SPHF decreased significantly (*p* < 0.05) until 6% DH, but the changes after that were insignificant (*p* > 0.05). Similarly, Yolandani, Liu, et al. (2024) noted that surface hydrophobicity tends to decrease in the proteolysis of soy protein with Alcalase until 15% DH. Additionally, the decreasing surface hydrophobicity of Flavourzyme hydrolysates was found to show insignificant changes after reaching 12% DH. Guan et al. (2018) also demonstrated that soy protein isolates had lower surface hydrophobicity as DH increased. do Evangelho et al. (2017) studied pepsin‐treated protein hydrolysates from common black bean and found that surface hydrophobicity decreased as DH increased. The decrease in the hydrophobicity of the proteins may be due to the burial of the hydrophobic groups in the interior of the protein cluster and the exposure of the more hydrophilic groups to the solvent during hydrolysis (do Evangelho et al. 2017).

#### 3.1.2 Color Properties

Color characteristics of SPI and SPH are presented in Table 1. The effect of hydrolysis on color characteristics was significant (*p* < 0.05). The L* value of SPI (65.97) significantly increased after hydrolysis with both Alcalase (70.69–71.77) and Flavourzyme (68.44–70.29), indicating that SPH were lighter in color (*p* < 0.05). SPHA showed a slightly higher L* value compared to SPHF in the same DH. The effect of DH on the L* value of SPHA was insignificant (*p* > 0.05). However, the L* values of SPHF exhibited a slightly decreasing trend with increasing DH (*p* < 0.05). Hydrolysis caused a decrease from 2.46 to the range of −0.07 to 0.76 in the a* value of SPI (*p* < 0.05). In both SPHA and SPHF samples, the highest b* value was observed at 10% DH. The total color difference in SPH was higher than the noticeable threshold, which was three units (Δ*E* > 3). These results suggest that hydrolysis causes protein samples to be lighter in color and appear less red. Moreover, the ΔE value of SPHA was higher than that of SPHF. This may be due to the difference in hydrolysis conditions (pH, incubation temperature, and time). Additionally, it was stated that the differences in color characteristics of protein samples may be related to variations in particle size (Dabbour et al. 2020).

**TABLE 1:** Color properties of safflower protein isolate and hydrolysates.

Sample	*L* *	*a* *	*b* *	Δ*E*
SPI	65.97 ± 0.38^E^	2.46 ± 0.02^A^	21.87 ± 0.04^CD^	—
SPHA‐2%	71.36 ± 0.50^A^	0.51 ± 0.01^CD^	21.53 ± 0.14^DE^	5.40 ± 0.51^AB^
SPHA‐4%	70.69 ± 0.35^ABC^	0.65 ± 0.07^BC^	21.03 ± 0.28^EF^	4.82 ± 0.29^ABC^
SPHA‐6%	71.77 ± 0.18^A^	0.27 ± 0.02^E^	20.69 ± 0.13^F^	5.93 ± 0.17^A^
SPHA‐8%	71.22 ± 0.60^AB^	−0.05 ± 0.02^F^	21.60 ± 0.09^DE^	5.29 ± 0.58^ABC^
SPHA‐10%	71.28 ± 0.38^AB^	−0.07 ± 0.02^F^	22.40 ± 0.12^C^	5.36 ± 0.38^AB^
SPHF‐2%	70.29 ± 0.36^ABCD^	−0.03 ± 0.01^F^	22.35 ± 0.06^C^	4.39 ± 0.35^ABC^
SPHF‐4%	69.42 ± 0.46^BCD^	0.54 ± 0.04^CD^	23.61 ± 0.05^B^	3.87 ± 0.43^BC^
SPHF‐6%	68.94 ± 0.36^CD^	0.46 ± 0.02^D^	23.72 ± 0.15^B^	3.52 ± 0.31^C^
SPHF‐8%	68.73 ± 0.09^D^	0.45 ± 0.01^D^	24.21 ± 0.04^AB^	3.62 ± 0.04^BC^
SPHF‐10%	68.44 ± 0.17^D^	0.76 ± 0.03^B^	24.77 ± 0.16^A^	3.82 ± 0.08^BC^

* Different capital letters in the same column are significantly different (*p* < 0.05).

#### 3.1.3 Bulk and Tapped Bulk Densities, Hausner Ratio, Carr Index, and Wettability

Powder properties, including bulk and tapped densities, Hausner ratio, Carr index, and wettability values of SPI and SPH are given in Table 2. The bulk density of SPI decreased to the range of 0.21–0.24 g/cm^3^ after hydrolysis with Alcalase (*p* < 0.05). Wani et al. (2015) found that black gram protein hydrolysates had significantly lower bulk density than protein isolates. Muhamyankaka et al. (2013) associated low bulk density with large particle size and low particle density. On the other hand, hydrolysis with Flavourzyme resulted in an increase in the bulk density of the samples (*p* < 0.05). Additionally, the bulk densities of the samples increased with increasing DH in both Alcalase and Flavourzyme hydrolysis. Singh et al. (2021) also observed that the bulk density of the rice bran protein isolates increased after the hydrolysis process, and this increase followed an increasing trend as DH increased. The authors attributed the high bulk density of the hydrolysates to small particles formed during hydrolysis, filling the spaces between larger peptides (Singh et al. 2021). Similar to bulk density results, the tapped density of SPHA was lower than that of SPHF (*p* < 0.05). These differences can be associated with the difference between particle size distribution and porosity of hydrolysates (Sarabandi et al. 2018).

**TABLE 2:** Powder properties of safflower protein isolate and hydrolysates.

Sample	Bulk density* (g/cm^3^)	Tapped density* (g/cm^3^)	Hausner ratio*	Carr index* (%)	Wettability* (s)
SPI	0.25 ± 0.01^CD^	0.43 ± 0.01^AB^	1.70 ± 0.03^A^	41.22 ± 0.99^A^	114.00 ± 1.73^A^
SPHA‐2%	0.22 ± 0.01^EF^	0.31 ± 0.01^DE^	1.40 ± 0.03^BCD^	28.55 ± 1.52^BC^	88.67 ± 2.33^B^
SPHA‐4%	0.21 ± 0.01^F^	0.30 ± 0.01^E^	1.39 ± 0.02^BCD^	28.19 ± 0.94^BC^	58.00 ± 1.53^DE^
SPHA‐6%	0.21 ± 0.01^F^	0.30 ± 0.01^E^	1.44 ± 0.01^B^	30.54 ± 0.29^B^	54.00 ± 1.15^E^
SPHA‐8%	0.24 ± 0.01^DE^	0.34 ± 0.01^D^	1.41 ± 0.01^BCD^	29.07 ± 0.58^BC^	51.67 ± 2.19^E^
SPHA‐10%	0.24 ± 0.01^DE^	0.34 ± 0.01^D^	1.43 ± 0.01^BC^	30.05 ± 0.63^B^	45.33 ± 1.20^E^
SPHF‐2%	0.27 ± 0.01^C^	0.38 ± 0.01^C^	1.39 ± 0.03^BCD^	27.77 ± 1.45^BC^	123.33 ± 3.38^A^
SPHF‐4%	0.31 ± 0.01^B^	0.40 ± 0.01^BC^	1.33 ± 0.01^D^	24.62 ± 0.38^C^	116.33 ± 3.76^A^
SPHF‐6%	0.31 ± 0.01^B^	0.42 ± 0.01^AB^	1.34 ± 0.01^CD^	25.38 ± 0.22^C^	75.67 ± 3.84^C^
SPHF‐8%	0.32 ± 0.01^AB^	0.42 ± 0.01^AB^	1.33 ± 0.01^D^	24.75 ± 0.51^C^	71.00 ± 2.52^C^
SPHF‐10%	0.34 ± 0.01^A^	0.45 ± 0.01^A^	1.33 ± 0.02^D^	24.75 ± 1.19^C^	68.67 ± 2.73^CD^

* Different capital letters in the same column are significantly different (*p* < 0.05).

Hausner ratio and Carr index are used to evaluate the cohesiveness and flowability of powders, respectively (Başyiğit et al. 2022). These parameters of SPI were 1.70% and 41.22%, respectively, indicating that SPI had high cohesiveness and bad flowability (Table 2). Hydrolysis resulted in significant decreases in the Hausner ratio and Carr index values of SPI (*p* < 0.05). The cohesiveness of SPHA was defined as “intermediate‐high” while that of SPHF was defined as “intermediate” Furthermore, SPH showed fair flowability due to Carr index values between 20% and 35% (Akyüz and Ersus 2021).

The wettability of SPI was found to be 114 s, and it was significantly decreased with hydrolysis Alcalase at 2%–10% HD and Flavourzyme at 6%–10% HD (*p* < 0.05; Table 2). There was a decrease in the wettability of SPHA and SPHF samples with DH increased (*p* < 0.05). Moreover, SPHA exhibited lower wettability values compared to SPHF at the same DH. The wettability of proteins may be affected by the surface area (Akyüz and Ersus 2021) and properties (Başyiğit et al. 2022). It was also stated that polar and nonpolar groups on the particle surface may affect the wetting time (Gomes and Kurozawa 2024).

### 3.2 Functional Properties

#### 3.2.1 Protein Solubility

**FIGURE 2:**
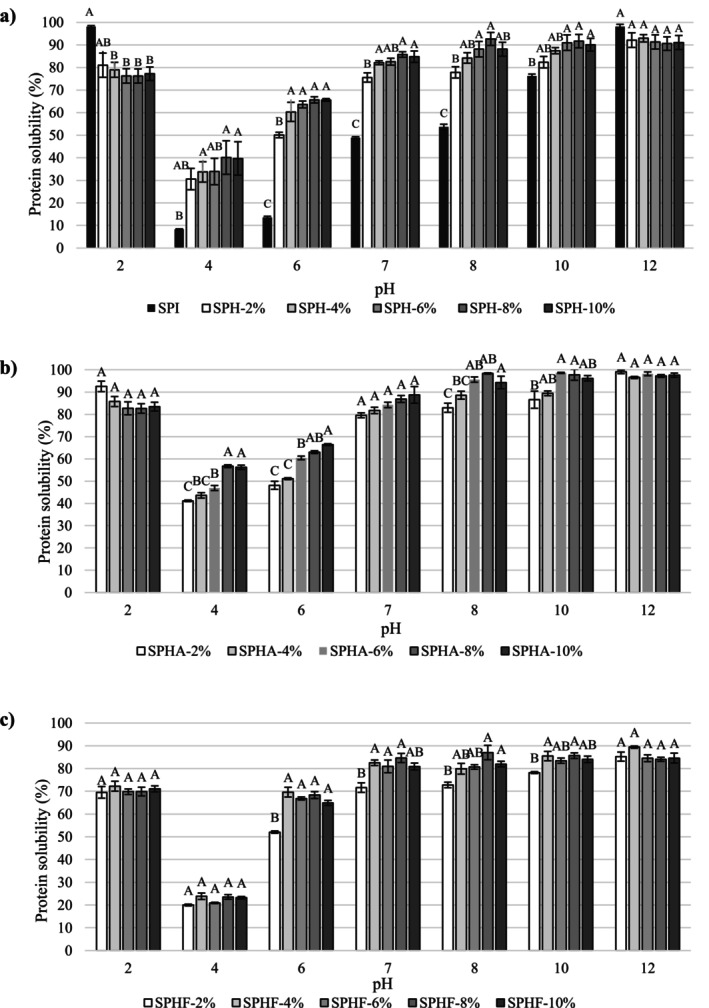
Protein solubility of safflower protein isolate and hydrolysates depending on pH values. *p* < 0.05 is statistically significant.

PS has an important role in protein function as it affects many properties of proteins, such as foaming, emulsion, and gelling (Noman et al. 2018). Figure 2 shows the PS of SPI and SPH in the pH range of 2.0–12.0. The PS profiles of the SPI were V‐shaped, and the highest PS was detected at both pH 2.0 and pH 12.0 (Figure 2a). SPH with lower DH (2%–4% DH) had a similar profile and showed the highest solubility at pH 12.0. However, SPHA with higher DH (6%–10% DH) reached the highest PS at pH 8 (higher than 94%), and the variation in PS at pH 8–12 was found to be insignificant (*p* > 0.05; Figure 2b). Moreover, SPHF with higher DH (6%–10%) attained the highest PS at pH 7, with its PS varying between 80.68% and 87.01% at pH 7–12 (*p* > 0.05; Figure 2c). Enzymatic hydrolysis with both Alcalase and Flavourzyme significantly improved the PS of SPI in the pH range of 4–10 (*p* < 0.05). The increase in PS of SPI with hydrolysis in the range of pH 4–7 is particularly important as the pH of food systems is often slightly acidic or neutral (Ghribi et al. 2015; Mune Mune 2015). SPI and SPH samples showed the lowest PS at pH 4, which is close to the pI value reported by Korkmaz (2024). At this pH, the PS of SPI was only 8.29%, and the PS of SPH was significantly higher than SPI (*p* < 0.05). Compared with SPHF, PS values were significantly higher in SPHA, and the highest value was found to be 56.71% in SPHA‐8%. When the pH increased from 4 to 6, there was an increase in the PS of SPHA in the range of 6.32%–13.49%, while the dramatic increase in the PS of SPHF was in the range of 32.06%–45.91%. The PS of SPH was determined to be in the range of 71.63%–88.74% at pH 7, and the lowest PS was found at 2% DH in both SPHA and SPHF (*p* < 0.05). Similarly, SPH with 2% DH showed the lowest PS value in both SPHA and SPHF at pH 8 (*p* < 0.05). Moreover, the PS of SPH significantly increased until 8% DH but then showed a slight decrease up to 4.55% (*p* < 0.05). These results agreed with the findings of Mune Mune (2015) on the pepsin‐hydrolyzed chickpea protein. High DH may promote the reveal of hidden hydrophobic groups, resulting in the formation of insoluble aggregates and subsequently reducing PS (Mune Mune 2015). Additionally, it was also confirmed that enzymatic hydrolysis resulted in an increase in PS of *Zanthoxylum* seed (Dong et al. 2024), peanut (Yadav et al. 2022), lentil (Thirulogasundar et al. 2024), chickpea (Ghribi et al. 2015; Thirulogasundar et al. 2024), and rice bran (Singh et al. 2021) proteins. The improvement of the solubility of SPI by hydrolysis may be due to the reduction in molecular size and the generation of smaller peptide molecules with polar sides that can bind more water (Noman et al. 2018; Thirulogasundar et al. 2024; Yadav et al. 2022). Furthermore, Singh et al. (2021) postulated that solubility negatively relates to the surface hydrophobicity of proteins or hydrolysate.

#### 3.2.2 Water Holding Capacity and Oil Binding Capacity

WHC, the ability of the protein to physically hold water against gravity, is an important characteristic of protein in foods like soup, custard, and dough as it helps to absorb water and maintain quality (Thirulogasundar et al. 2024). There was a significant difference in WHC of SPI and SPH (*p* < 0.05; Table 3). Hydrolysis with both Alcalase and Flavourzyme caused a significant decrease in WHC of SPI (*p* < 0.05). Yolandani, Ma, et al. (2024) also found that the WHC of soy protein isolate decreased from 4.44 to 1.03 g/g after hydrolysis with bromelain at 12% DH. Similarly, a significant decrease in WHC of chickpea protein isolate was reported with trypsin hydrolysis (5%–20% DH; Thirulogasundar et al. 2024). SPHF demonstrated a significantly higher WHC compared to SPHA at the same DH (*p* < 0.05). It may be attributed to the different molecular weights of hydrolysates produced using different enzymes (Cumby et al. 2008). Additionally, a decrease in the WHC of the samples was observed as DH increased for both Alcalase and Flavourzyme hydrolysis. A similar trend was reported for the hydrolysis of faba bean protein with trypsin in the range of 5%–20% DH (Sareen et al. 2023). Supporting the results of this study, it was demonstrated that WHC has an inverse association with PS (Özdemir et al. 2022). Accordingly, reductions in molecular size through hydrolysis can lead to an increase in solubility, which may result in a decrease in WHC (Noman et al. 2018).

**TABLE 3:** Functional properties of safflower protein isolate and hydrolysates.

Sample	WHC* (g/g)	OBC* (g/g)	FC* (%)	FS* (%)	EC* (%)	ES* (%)	LGC (%)
SPI	1.56 ± 0.03^A^	2.55 ± 0.12^ABC^	31.67 ± 1.67^C^	82.22 ± 1.11^A^	55.73 ± 2.33^CD^	85.63 ± 1.22^A^	10.00 ± 0.00
SPHA‐2%	0.88 ± 0.02^BC^	2.81 ± 0.12^A^	115.83 ± 4.64^A^	18.33 ± 1.67^D^	58.22 ± 0.47^BC^	25.77 ± 0.21^C^	24.00 ± 0.00
SPHA‐4%	0.68 ± 0.03^F^	2.68 ± 0.11^AB^	120.83 ± 4.17^A^	22.50 ± 1.91^D^	63.67 ± 0.17^ABC^	23.56 ± 0.06^C^	26.00 ± 0.00
SPHA‐6%	0.69 ± 0.01^EF^	2.64 ± 0.05^AB^	119.17 ± 3.63^A^	19.17 ± 0.83^D^	65.67 ± 3.97^AB^	23.02 ± 1.48^C^	28.00 ± 0.00
SPHA‐8%	0.66 ± 0.01^F^	2.33 ± 0.05^ABC^	123.33 ± 1.67^A^	27.08 ± 2.08^D^	68.42 ± 1.96^A^	21.96 ± 0.65^C^	28.00 ± 0.00
SPHA‐10%	0.30 ± 0.01^G^	2.34 ± 0.11^ABC^	120.83 ± 4.17^A^	18.75 ± 0.72^D^	61.80 ± 1.05^ABC^	24.28 ± 0.41^C^	42.00 ± 0.00
SPHF‐2%	0.96 ± 0.02^B^	2.36 ± 0.11^ABC^	38.33 ± 0.83^C^	69.44 ± 2.78^AB^	62.42 ± 0.22^ABC^	24.03 ± 0.09^C^	10.00 ± 0.00
SPHF‐4%	0.81 ± 0.02^CD^	2.37 ± 0.02^ABC^	68.33 ± 4.41^B^	60.22 ± 5.17^BC^	63.08 ± 1.47^ABC^	23.80 ± 0.55^C^	12.00 ± 0.00
SPHF‐6%	0.76 ± 0.01^DE^	2.30 ± 0.10^BC^	78.33 ± 3.33^B^	50.79 ± 2.54^C^	48.92 ± 1.54^DE^	30.73 ± 0.99^B^	12.00 ± 0.00
SPHF‐8%	0.74 ± 0.01^DEF^	2.26 ± 010^BC^	80.00 ± 5.00^B^	52.38 ± 4.15^C^	46.25 ± 0.88 ^E^	32.46 ± 0.63^B^	16.00 ± 0.00
SPHF‐10%	0.68 ± 0.02^EF^	2.15 ± 0.09^C^	70.83 ± 4.17^B^	48.22 ± 0.97^C^	44.33 ± 1.45^E^	33.91 ± 1.10^B^	16.00 ± 0.00

* Different capital letters in the same column are significantly different (*p* < 0.05).

OBC, which evaluates the ability of hydrophobic side chains in proteins to bind with oil, influencing flavor retention and mouthfeel in food (Thirulogasundar et al. 2024). SPI had an OBC value of 2.55 g/g, and the effect of hydrolysis on OBC was significant (*p* < 0.05; Table 3). On the other hand, SPHA showed slightly higher OBC than SPHF at the same DH. Many studies showed that the different enzymes used for the hydrolysis of soybean (Islam et al. 2022), faba bean (Eckert et al. 2019), and pumpkin protein (Muhamyankaka et al. 2013) resulted in different OBC values. In addition, increasing DH in both Alcalase and Flavourzyme hydrolysis had a decreasing effect on the OBC of SPH. It was stated that changes in the secondary structure, surface area (Yadav et al. 2022), molecular size (Noman et al. 2018), and hydrophobicity (Thirulogasundar et al. 2024) that occur with enzymatic hydrolysis may affect OBC.

#### 3.2.3 Foaming Capacity and Foam Stability

The foam structure is formed by the unfolding of proteins to form an interfacial layer that suspends air bubbles and prevents them from collapsing (Wani et al. 2015). Foam properties, including FC and FS, are key quality attributes for proteins used in food applications such as beverages, ice cream, mousses, cakes, and toppings (Thirulogasundar et al. 2024; Wani et al. 2015). SPI displayed an FC value of only 31.67%, which was significantly lower than the FC of all SPH (*p* < 0.05; Table 3). Yadav et al. (2022) also observed that peanut proteins hydrolyzed with different enzymes had higher FC compared to the non‐hydrolyzed sample. This increase in FC may be due to the presence of more proteins that can be adsorbed at the interface due to the increase in the solubility of the samples as a result of hydrolysis (Eckert et al. 2019; Ren et al. 2017). The FC of SPHA was significantly higher than SPHF (*p* < 0.05). The possible reason for this is that SPHA may have a higher electrostatic repulsion between small molecule peptides at the interface (Dong et al. 2024). However, no significant effect of DH on SPHA was found (*p* > 0.05). Likewise, there was no observed significant difference in the FC of SPHF after 2% DH (*p* > 0.05). These results agreed with the findings of Mune Mune (2015), who reported that pepsin hydrolysis significantly increased the FC of chickpea protein; however, the effect of DH (5% and 10%) on the FC of samples was insignificant. García Arteaga et al. (2020) also stated that the FC of pea protein displayed no significant differences depending on DH. The highest FS was observed in SPI at 82.22%, and hydrolysis resulted in a significant decrease in the FS of SPH (*p* < 0.05). This result is consistent with the studies conducted on sunflower (Ren et al. 2017), *Zanthoxylum* seed (Dong et al. 2024), lentil (Thirulogasundar et al. 2024), cowpea (Mune Mune 2015), and rice bran (Singh et al. 2021) proteins. Compared to SPHF, SPHA exhibited lower FS values (*p* < 0.05). Additionally, there was no significant difference between the FS of SPHA samples depending on the DH (*p* > 0.05). Ren et al. (2017) also declared that the FS of sunflower protein hydrolysates with 10%–30% DH changed insignificantly. On the other side, the FS of SPHF displayed a significant decreasing trend between 2% and 6% DH (*p* < 0.05), yet it displayed insignificant changes after 6% DH (*p* > 0.05). It was pointed out that large protein structures form more stable foam structures. Accordingly, increasing the amount of small peptides that cannot form a stable foam structure through hydrolysis may be the reason for the decrease in FS (Mune Mune 2015). Moreover, lower FS may be related to lower surface hydrophobicity (Yadav et al. 2022) and higher solubility (Dong et al. 2024; Singh et al. 2021) of SPH.

#### 3.2.4 Emulsion Capacity and Emulsion Stability

The emulsion structure is formed depending on the ability of proteins to diffuse, adsorb, rearrange, interact, and create elastic films at the water–oil interface (Wouters et al. 2016). The EC was significantly affected by hydrolysis, as shown in Table 3, and the EC of samples ranged between 44.33% and 68.42% (*p* < 0.05). SPHA exhibited higher EC than SPI; the EC of samples increased as DH increased from 2% to 8% (*p* < 0.05). An increase in EC was also reported for chickpea proteins hydrolyzed by Alcalase (Ghribi et al. 2015) and pepsin (Mune Mune 2015). The authors attributed this increase to the rapid migration and adsorption of proteins to the water–oil interface to create a film due to increased solubility (Ghribi et al. 2015; Mune Mune 2015). On the other hand, the EC of SPHF exhibited a significant decrease when DH increased (*p* < 0.05). Similar decreases were reported for the EC of sunflower (Ren et al. 2017), coconut (Thaiphanit et al. 2016), and Black gram seed (Wani et al. 2015). It was hypothesized that the weakening of EC of samples is due to impairing the amphipathic character of peptides (Ren et al. 2017; Singh et al. 2021). Hydrolysis dramatically decreased the ES of SPI from 85.63% to 21.96%–33.91% (*p* < 0.05). Small peptides cannot form stable emulsions because their charge repulsion may prevent them from easily aggregating to create a fat globule membrane (Ghribi et al. 2015; Liu et al. 2010; Singh et al. 2021). Moreover, it was reported that this result could be attributed to the decrease in surface hydrophobicity (do Evangelho et al. 2017; Wang et al. 2021).

#### 3.2.5 Least Gelation Concentration

The gel structure occurs when proteins form a three‐dimensional cross‐linked network that can bind water. When critical concentration is reached, this structure is formed and maintained. The gelling properties of proteins are important for a variety of foods, including meat alternatives, tofu, yogurt, and desserts (Vogelsang‐O'Dwyer et al. 2023). As seen in Table 3, hydrolysis of both Alcalase and Flavourzyme led to an increase in the LGC of SPI. The higher values of LGC were observed in SPHA compared to SPHF. Additionally, higher concentrations of SPH were able to form a gel structure as DH increased. Many studies confirmed that enzymatic hydrolysis had a negative effect on the gelling properties of soy protein (Ashaolu 2020; Wouters et al. 2016). This may be related to lower molecular size, which limits the ability to form a gel network, and lower hydrophobicity and leads to a lower extent of protein–protein interaction (Ashaolu 2020; Wouters et al. 2016).

### 3.3 Antioxidant Activity

**FIGURE 3:**
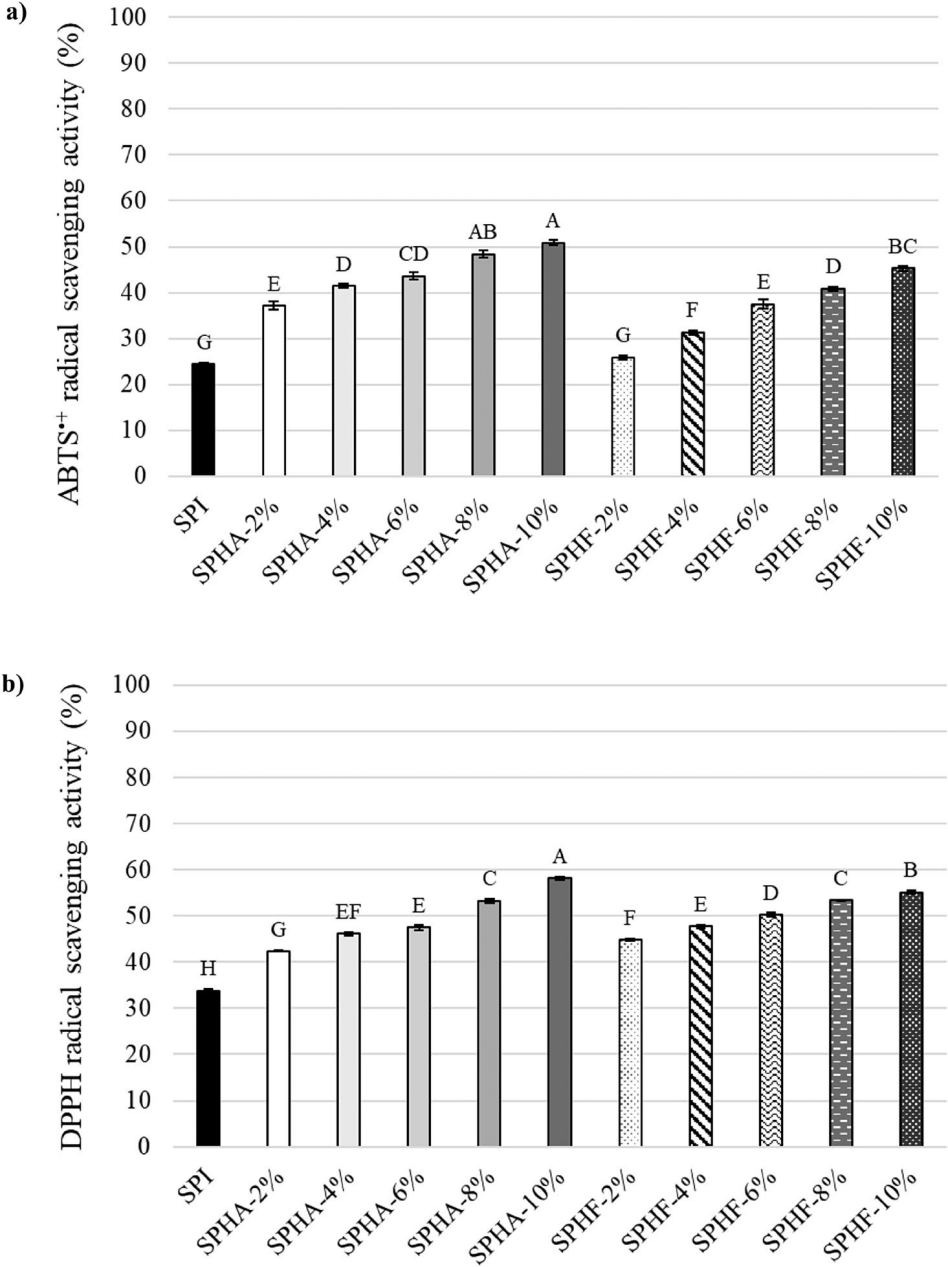
Antioxidant activity of safflower protein isolate and hydrolysates measured by ABTS^•+^ radical scavenging activity (a) and DPPH radical scavenging activity (b). *p* < 0.05 is statistically significant.

The antioxidant activity of SPI and SPH determined by measured ABTS^•+^ (a) and DPPH (b) radical scavenging activities is represented in Figure 3. In the ABTS^•+^ radical assay, antioxidant activity is evaluated by reacting the radical to form an ABTS^•+^ radical complex (Islam et al. 2022). As clearly shown in Figure 3a, the lowest ABTS^•+^ radical scavenging activities were observed in SPI among all protein concentrations (*p* < 0.05). Additionally, SPHA showed higher ABTS^•+^ radical scavenging activities compared to SPHF. The ABTS^•+^ radical scavenging activity of SPHA‐10% was the highest among all samples (*p* < 0.05). Moreover, a positive relationship was observed between the ABTS^•+^ scavenging activities of SPH and DH. Wang et al. (2021) hydrolyzed tree peony seed protein using different enzymes, including Alcalase, Neutrase, Papain, Protamex, and Flavourzyme. It was found that the ABTS^•+^ radical scavenging activity of tree peony seed protein increased after hydrolysis, and the Alcalase hydrolysate had the strongest ABTS^•+^ radical scavenging activity among all hydrolysates (Wang et al. 2021). Similarly, when camelina protein isolate and its hydrolysates were compared by Ngo and Shahidi (2021), it was found that Alcalase hydrolysate exhibited the highest ABTS^•+^ radical scavenging activity, followed by Flavourzyme hydrolysate and protein isolate. These results may be related to the ability of small molecular weight peptides to effectively adsorb radicals (Islam et al. 2022). Additionally, a decrease in molecular size can enhance solubility, resulting in more interactions with ABTS^•+^ hydrophilic radicals (Rezvankhah et al. 2022).

DPPH radical scavenging activity is widely used to evaluate the antioxidative properties of hydrogen atom donors and free radical scavengers. It is affected by many parameters, such as molecular size, amino acid composition, and protease used for hydrolysis (Ngo and Shahidi 2021). The DPPH radical scavenging activities of SPH were significantly higher than those of SPI (*p* < 0.05). These findings support previously reported studies on camelina (Ngo and Shahidi 2021), tree peony seed (Wang et al. 2021), and lentil (Rezvankhah et al. 2022) protein hydrolysates exhibiting higher DPPH radical scavenging activity than protein isolates. The DPPH radical scavenging activities of SPH increased as DH increased (*p* < 0.05). On the other hand, similar to the ABTS^•+^ radical scavenging activity results, the highest DPPH radical scavenging activity was found in SPHA‐10%. These results may be explained by the higher chance of small peptides adsorbing oxidative agents (Rezvankhah et al. 2022).

## 4 Conclusions

In this study, the effect of enzymatic hydrolysis performed with Alcalase and Flavourzyme at five different DH levels on the powder, functional, and antioxidant properties of SPI was evaluated. Alterations in particle size and surface properties due to hydrolysis led to a decrease in the cohesiveness of the SPH and enhanced their flowability. On the other hand, it was found that SPH had lower surface hydrophobicity with higher solubility than SPI. Besides, the WHC of proteins had an inverse association with PS. Additionally, the WHC and OBC of SPH showed a decreasing trend with increasing DH. SPHA demonstrated higher FC and EC compared to SPHF, with SPHA‐8% showing the highest FC and EC among SPI and SPH. However, poorer foam and emulsion structure stability was observed in SPH compared to SPI due to the decrease in peptide molecule size and surface hydrophobicity and increased solubility after hydrolysis. Furthermore, SPH was able to form a gel structure at higher concentrations than SPI, and the LGC of SPH also increased as DH increased. On the other hand, SPH showed more ABTS^•+^ and DPPH radical scavenging abilities than SPI, and the ABTS^•+^ and DPPH radical scavenging activities of SPH increased as DH increased. Overall, the results revealed that enzymatic hydrolysis significantly improved the powder, functional, and antioxidant properties of SPI, depending on the types of enzymes and DH.

In conclusion, SPH has a remarkable potential for use as a functional and antioxidant ingredient in the food and pharmaceutical industries. These results contribute to the literature by providing a deeper understanding of how enzymatic hydrolysis modulates the physicochemical and functional properties of SPI, emphasizing its potential applications in product development. Future studies can explore the optimization of hydrolysis conditions to further enhance specific functionalities, investigate the stability of SPH in various food matrices, and evaluate its bioavailability and health benefits.

## Author Contributions

**Fatma Korkmaz:** formal analysis (equal), investigation (equal), methodology (lead), project administration (lead), validation (equal), writing – original draft (equal), writing – review and editing (equal). **Ceren Mutlu:** formal analysis (equal), investigation (equal), validation (equal), writing – original draft (equal), writing – review and editing (equal).

## Ethics Statement

No ethical approval was required for the study.

## Conflicts of Interest

The authors declare no conflicts of interest.

## Supporting information


Figure S1.

## Data Availability

The data that support the findings of this study are available on request from the corresponding author.
