# Supplementary figures and images for: Safflower Protein Hydrolysates: Physicochemical, Functional Properties and Antioxidant Activities
Source: Food Sci Nutr. 2025 May 11;13(5):e70258. doi: 10.1002/fsn3.70258 (PMC12066243; doi:10.1002/fsn3.70258)

**Fig S1.** Photographs of the samples

| 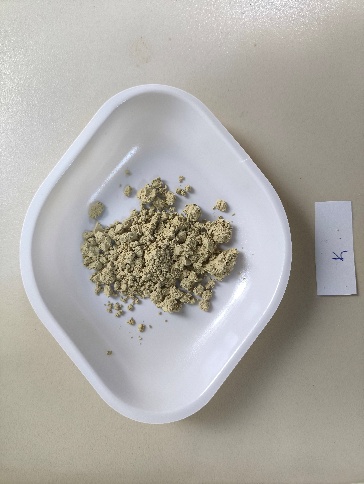 SPI | 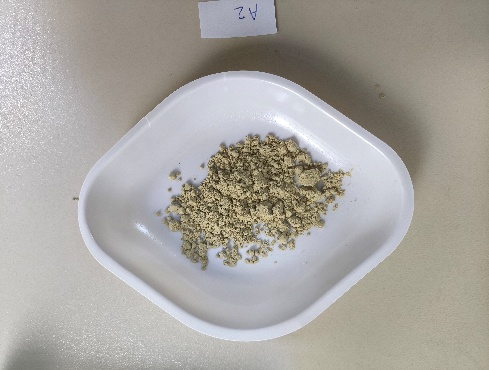 | 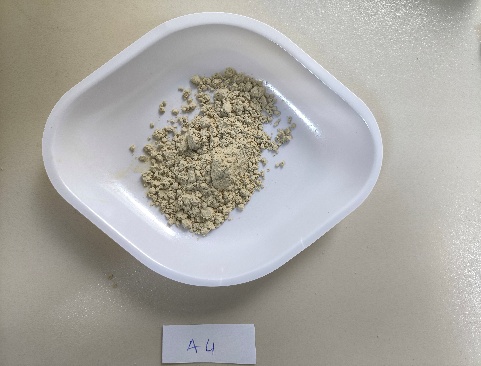 | 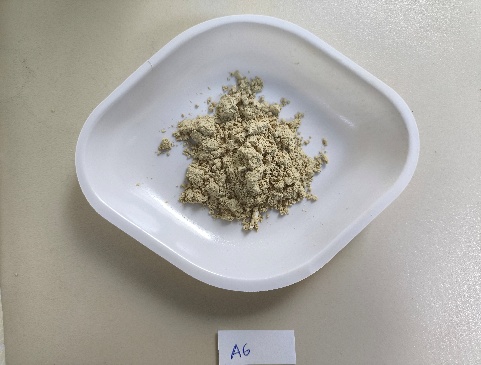 | 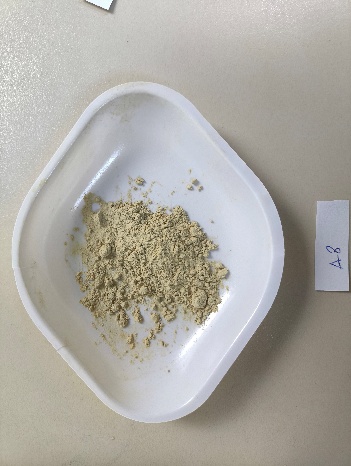 | 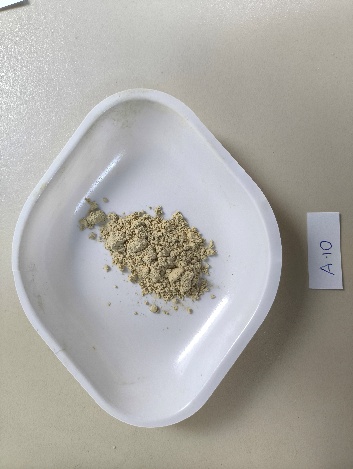 |
| --- | --- | --- | --- | --- | --- |
| SPHA-2% | SPHA-4% | SPHA-6% | SPHA-8% | SPHA-10% |
| 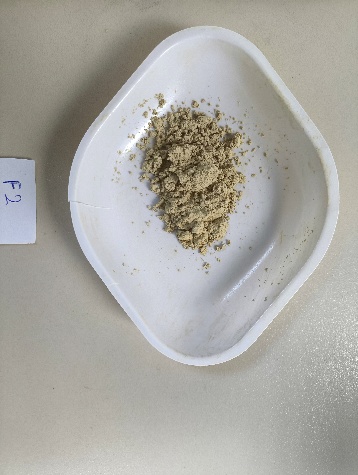 | 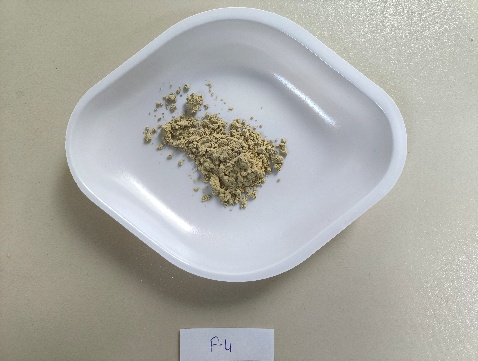 | 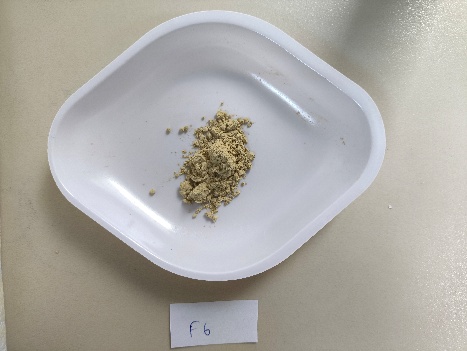 | 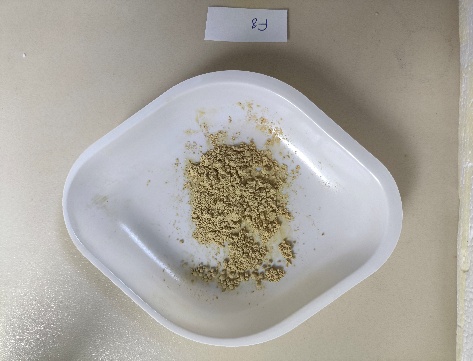 | 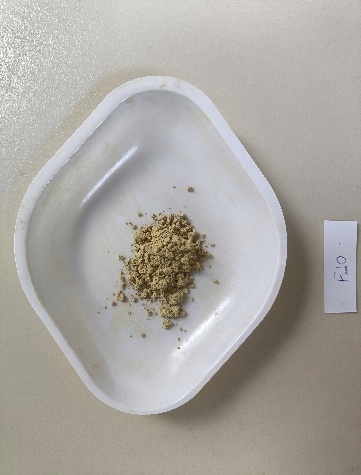 |
| SPHF-2% | SPHF-4% | SPHF-6% | SPHF-8% | SPHF-10% |

Supplement: Supplementary file 1 — Figure S1. [file FSN3-13-e70258-s001.docx]
